# *In vivo* tracking of phosphoinositides in *Drosophila* photoreceptors

**DOI:** 10.1242/jcs.180364

**Published:** 2015-12-01

**Authors:** Roger C. Hardie, Che-Hsiung Liu, Alexander S. Randall, Sukanya Sengupta

**Affiliations:** Department of Physiology Development and Neuroscience, Cambridge University, Cambridge CB2 3EG, UK

**Keywords:** PIP_2_, Pleckstrin homology domain, Phototransduction, Fluorescent probes, GFP, Voltage-sensitive phosphatase

## Abstract

In order to monitor phosphoinositide turnover during phospholipase C (PLC)-mediated *Drosophila* phototransduction, fluorescently tagged lipid probes were expressed in photoreceptors and imaged both in dissociated cells, and in eyes of intact living flies. Of six probes tested, Tb^R332H^ (a mutant of the Tubby protein pleckstrin homology domain) was judged the best reporter for phosphatidylinositol (4,5)-bisphosphate [PtdIns(4,5)*P*_2_], and the P4M domain from *Legionella* SidM for phosphatidylinositol 4-phosphate (PtdIns4*P*). Using accurately calibrated illumination, we found that only ∼50% of PtdIns(4,5)*P*_2_ and very little PtdIns4*P* were depleted by full daylight intensities in wild-type flies, but both were severely depleted by ∼100-fold dimmer intensities in mutants lacking Ca^2+^-permeable transient receptor potential (TRP) channels or protein kinase C (PKC). Resynthesis of PtdIns4*P* (*t*½ ∼12 s) was faster than PtdIns(4,5)*P*_2_ (*t*½ ∼40 s), but both were greatly slowed in mutants of DAG kinase (*rdgA*) or PtdIns transfer protein (*rdgB*). The results indicate that Ca^2+^- and PKC-dependent inhibition of PLC is required for enabling photoreceptors to maintain phosphoinositide levels despite high rates of hydrolysis by PLC, and suggest that phosphorylation of PtdIns4*P* to PtdIns(4,5)*P*_2_ is the rate-limiting step of the cycle.

## Abstract (precis)

**Highlighted Article:** A range of genetically targeted lipid probes were used to track and quantify phosphoinositide turnover in the photoreceptors of completely intact living flies, with a precision unsurpassed in other systems.

## INTRODUCTION

Phototransduction in *Drosophila* is mediated by a G-protein-coupled phospholipase C (PLC) cascade and is an influential model for phosphoinositide signalling (Hardie, 2012; Hardie and Juusola, 2015; Katz and Minke, 2009; Montell, 2012; Yau and Hardie, 2009). The electrical response to light is mediated by two Ca^2+^-permeable cation channels: transient receptor potential (TRP) and TRP-like (TRPL) (Hardie and Minke, 1992; Montell and Rubin, 1989; Phillips et al., 1992). Like their mammalian TRP channel homologues, both are activated downstream of PLC. The channels are localised in a light-guiding ‘rhabdomere’ (a rod-like stack of ∼30,000 microvilli) along with other components of the phototransduction cascade, including rhodopsin, Gq protein and PLC (encoded by *norpA*) (Hardie, 2012; Huber et al., 1996). How hydrolysis of phosphatidylinositol (4,5)-bisphosphate [PtdIns(4,5)*P*_2_] by PLC activates TRP and TRPL channels remains debated. Recent evidence has suggested a combinatorial mechanism requiring both a reduction in PtdIns(4,5)*P*_2_ and acidification mediated by the protons released by the PLC reaction (Huang et al., 2010). The depletion of PtdIns(4,5)*P*_2_ might act mechanically by cleaving its large and highly charged headgroup from the membrane, leading to a physical contraction of the microvillar membrane (Hardie and Franze, 2012).

PtdIns(4,5)*P*_2_ and other phosphoinositides are vital regulators of numerous cellular functions (Balla, 2013; Di Paolo and De Camilli, 2006; Hilgemann et al., 2001; Payrastre et al., 2001; Rohacs, 2009; Yin and Janmey, 2003), hence their metabolism plays crucial roles in many cells. Previous studies have indicated that light-induced PtdIns(4,5)*P*_2_ hydrolysis by PLC in *Drosophila* is initially very rapid, but then abruptly inhibited by Ca^2+^ influx through the TRP channels (Gu et al., 2005; Hardie et al., 2004, 2001). In these experiments, PtdIns(4,5)*P*_2_ was monitored using a genetically encoded electrophysiological biosensor, the Kir2.1 K^+^ channel, which is activated by PtdIns(4,5)*P*_2_ (Hilgemann and Ball, 1996). Although a powerful tool, the use of Kir2.1 as a biosensor necessitates patch-clamp recordings, which are invasive, technically demanding and have a limited lifetime. Another class of tools used to monitor phosphoinositide turnover, are fluorescently tagged phosphoinositide-binding domains. The first and most widely used of these is the pleckstrin homology (PH) domain from PLCδ1 (Stauffer et al., 1998; Varnai and Balla, 1998). However, interpretation is complicated because PLCδ1-PH binds inositol (1,4,5)-trisphosphate (Ins*P*_3_) as well as PtdIns(4,5)*P*_2_ (e.g. Hirose et al., 1999; Nash et al., 2001; Szentpetery et al., 2009). More recently, PtdIns(4,5)*P*_2_ probes with negligible affinity for InsP_3_, based on the Tubby protein have been developed (Hughes et al., 2007; Quinn et al., 2008), and probes with affinity for other phosphoinositide species are also available (Balla et al., 2009; Balla and Varnai, 2009; Szentpetery et al., 2009; Yu et al., 2004).

In the present study, we expressed a range of fluorescently tagged lipid probes in *Drosophila* photoreceptors. We used them to monitor phosphoinositide turnover, both in dissociated cells and in intact flies by exploiting the optics of the compound eye, which allow imaging of the rhabdomeres in live animals over many hours. Combined with the ability to activate the cascade with precisely calibrated illumination, and expression of a voltage-sensitive phosphatase allowing depletion of PtdIns(4,5)*P*_2_ by conversion into phosphatidylinositol 4-phosphate (PtdIns4*P*) (Iwasaki et al., 2008; Murata et al., 2005), we used these probes to provide quantitative insight into phosphoinositide turnover under physiologically relevant illumination in both wild-type flies and a variety of mutant backgrounds.

## RESULTS

A range of fluorescently tagged lipid binding domains were expressed in *Drosophila* photoreceptors under control of the rhodopsin (*ninaE*, also known as Rh1) or *trp* promoters. These included GFP-tagged PLCδ1 PH (hereafter referred to as PH–GFP), which binds to both PtdIns(4,5)*P*_2_ and Ins(1,4,5)*P*_3_ (Hirose et al., 1999; Stauffer et al., 1998; Szentpetery et al., 2009; Varnai and Balla, 1998); a PH domain from the C-terminal of the Tubby protein (YFP tagged), which has a high affinity for PtdIns(4,5)*P*_2_ but negligible affinity for Ins*P*_3_ (Santagata et al., 2001); a point mutation of the same construct, Tb^R332H^, with reduced affinity for PtdIns(4,5)*P*_2_ (Quinn et al., 2008); OSH2 (GFP tagged), which binds both PtdIns4*P* and PtdIns(4,5)*P*_2_ with similar affinities; OSH1 (GFP tagged) which also binds PtdIns4*P* and PtdIns(4,5)*P*_2_, but with a ∼3-fold lower affinity than OSH2 (Yu et al., 2004), and the P4M domain (GFP tagged) of *Legionella* SidM, which binds to PtdIns4*P* but not PtdIns(4,5)*P*_2_ (Brombacher et al., 2009; Hammond et al., 2014). In preliminary experiments, the wild-type Tubby PH domain translocated much more sluggishly than the other probes, suggesting its affinity was too high to be a useful indicator of PtdIns(4,5)*P*_2_ turnover in the photoreceptors, and it was not used further.

**Fig. 1.**
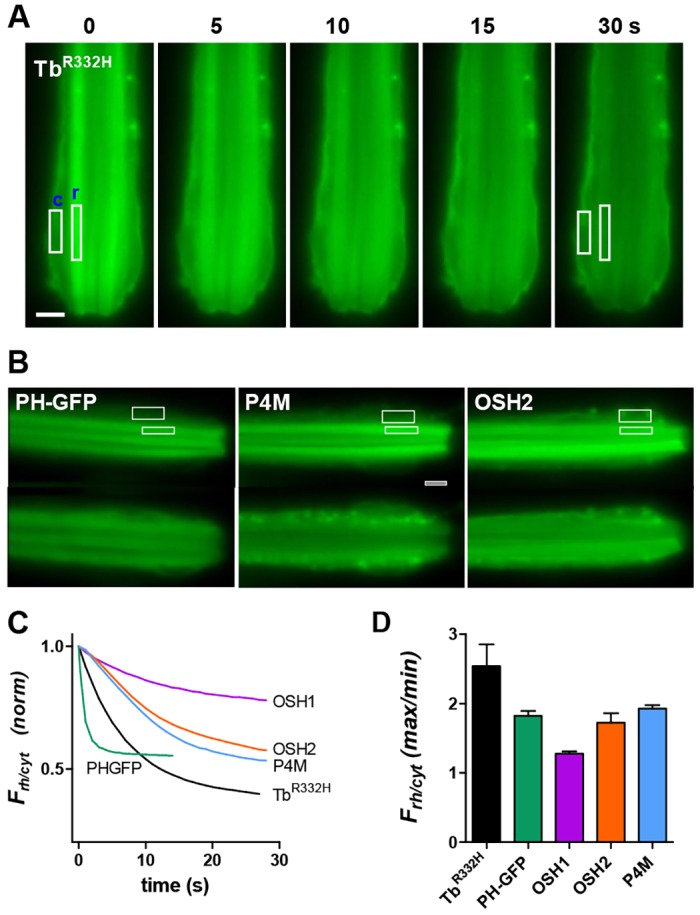
**Translocation of fluorescent probes in dissociated ommatidia.** (A) Fluorescent images from initially dark-adapted dissociated ommatidium expressing Tb^R332H^ immediately at onset of blue excitation (*t*=0) and 5, 10, 15 and 30 s later. Fluorescence was initially strongest in the rhabdomeres (r), but rapidly translocated to cell body and plasma membrane (c). (B) Fluorescence from dissociated ommatidia of flies expressing, PLCδ1-PH–GFP (PH–GFP), P4M and OSH2. The top image in each pair is at time=0; the bottom image is 30 s later. Following translocation PH-GFP localised to cytosol; P4M and OSH2 also localised to endomembranes. Scale bar: 5 µm. White boxes in A and B show typical ROIs used for measurement. (C) Timecourse of translocation measured as the normalised ratio of fluorescence from ROIs (e.g. boxes in A and B) in rhabdomere and cytosol (*F*_rh/cyt_). Each trace is the mean of measurements from 5–12 ommatidia. (D) Extent of fluorescence decay (*F*_rh/cyt_ max/min) expressed as ratio between *F*_rh/cyt_ at time zero and after 30 s illumination (mean±s.e.m., *n*=5–12).

In principle, expression of PtdIns(4,5)*P*_2_-binding proteins in the microvilli might affect phototransduction, for example, by sequestering PtdIns(4,5)*P*_2_. However, light responses in whole-cell patch clamp recordings from photoreceptors expressing the probes showed no significant differences from wild-type photoreceptors, with normal response kinetics, absolute sensitivity and single-photon responses (Fig. S1). Morphology at the light-microscope level was also not noticeably affected (e.g. Fig. 1) and cell capacitances, which provide a measure of the area of microvillar membrane, were indistinguishable from wild type (Fig. S1).

To visualise the subcellular localisation of the probes, dissociated ommatidia were imaged with conventional epi-fluorescence microscopy. Because the blue excitation light is a saturating stimulus for the photoreceptors, only the first image frame represents the dark-adapted situation, and any subsequent dynamic redistribution reflects the response to the blue excitation. In dark-adapted photoreceptors, all probes were initially well localised to the microvillar rhabdomeres, visible as brightly fluorescing rod-like tracks towards the centre of the ommatidia (Fig. 1). With little delay, fluorescence rapidly decreased in the rhabdomeres, and increased in cell body regions, reflecting translocation of the probes as PtdIns(4,5)*P*_2_ and/or PtdIns4*P* was depleted by light-activated PLC (Movies 1–3). The different probes translocated with subtly different patterns: PH–GFP accumulated diffusely in the cytosol, whereas Tb^R332H^ accumulated mainly on non-microvillar plasma membrane. OSH2, OSH1 and P4M also accumulated in discrete regions within the cell, presumably representing intracellular membranes (e.g. Golgi or the endosomes) as well as some mobile vesicles. These patterns are consistent with the reported affinities of the different probes. Thus only PH–GFP also binds to (InsP_3_), which would be expected to diffuse freely into the cytosolic aqueous phase. Tb^R332H^ binds specifically to PtdIns(4,5)*P*_2_, which is expected to have a predominantly plasma membrane localisation. OSH1, OSH2 and P4M bind to PtdIns4*P*, which is known to be localised to Golgi, plasma membrane and motile vesicles in mammalian cells (Hammond et al., 2014).

To quantify the signals, intensity was measured in regions of interest (ROIs) in the rhabdomere and the cytosol (Fig. 1), and the relative rhabdomere fluorescence (*F*_rh_/*F*_cyt_, hereafter *F*_rh/cyt_) plotted as a function of time. *F*_rh/cyt_ decayed with time constants of ∼7 s for Tb^R332H^, and ∼10–15 s for P4M, OSH2 and OSH1. PH–GFP translocated remarkably rapidly with a time constant of ∼1 s (Fig. 1C). In terms of the extent of translocation, Tb^R332H^ showed the greatest reduction in *F*_rh/cyt_ (∼2.5-fold), with OSH2, P4M and PH–GFP all showing ∼2-fold reduction. Although very reproducible, the OSH1 signal showed only a relatively minor (1.3-fold) reduction and was not used systematically for further experiments (Fig. 1D).

A complication inherent in any fluorescent study involving photoreceptors is that the excitation light itself is usually a supersaturating stimulus, photoisomerising a large fraction of the visual pigment rhodopsin (R) to its active metarhodopsin (M) state. In microvillar opsins, such as those in *Drosophila*, the two states (R and M) are thermostable and photo-interconvertible, and therefore exist in a photoequilibrium determined by their absorption spectra and the spectral content of illumination (Minke and Kirschfeld, 1979). Given that R absorbs maximally at 480 nm and M at 570 nm, the blue excitation light preferentially converts R into M, creating a photoequilibrium with ∼70% M and 30% R, which is reached within less than 100 ms (see Fig. S4) and then maintained indefinitely (many hours), or until M is photo-reisomerised into R by absorption of long-wavelength light. The active M state continues to activate the cascade (through G-proteins) until it is inactivated by binding to arrestin. However, because arrestin molecules are in limited supply (approximately one-third of the number of visual pigment molecules), there are insufficient to bind to and inactivate this amount (70%) of M. This results in maintained saturating activation of the phototransduction cascade and a so-called prolonged depolarising afterpotential (PDA), which lasts for many hours (Dolph et al., 1993; Minke, 1979). Apparently, in this PDA state, PtdIns(4,5)*P*_2_ and PtdIns4*P* in the rhabdomeres are largely depleted and remain so indefinitely, because even after prolonged periods in the dark, there was little if any recovery of fluorescence in the rhabdomeres by any of the probes either in dissociated ommatidia or in intact animals using the deep pseudopupil (see below and Fig. 4B). However, if M was photoreisomerised to the R state by long-wavelength (a 640 nm LED) light, substantial fractions of all probes returned to the rhabdomeres within 1 or 2 min in the dark, indicating resynthesis of PtdIns4*P* and PtdIns(4,5)*P*_2_ (see below).

### Deep pseudopupil measurements

**Fig. 2.**
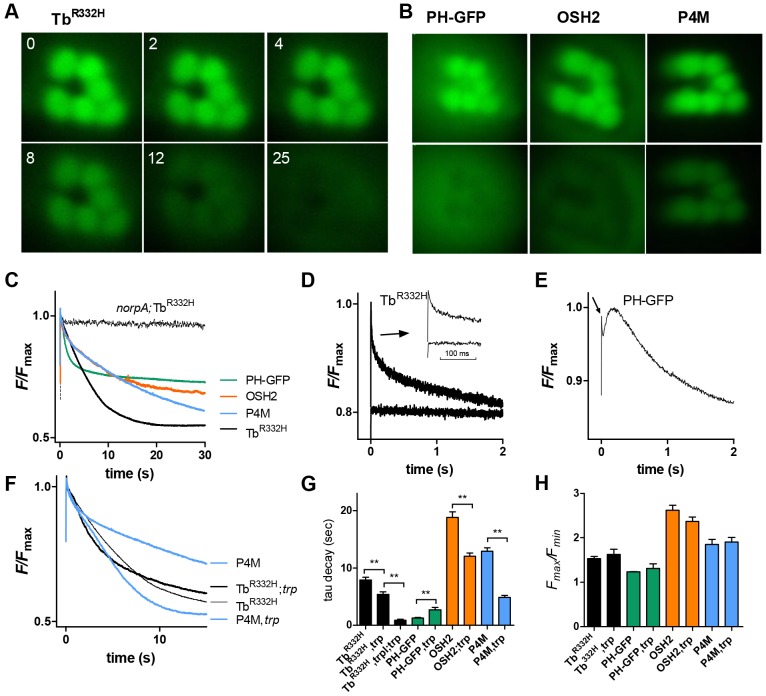
**Timecourse of translocation from DPP of intact flies.** (A) Images of DPP fluorescence in flies expressing Tb^R332H^ taken at 0, 2, 4, 8, 12 and 25 s after onset of blue excitation in an initially dark-adapted fly. (B) DPP fluorescence in flies expressing PH–GFP, OSH2 and P4M. The top image in each pair was taken at *t*=0; lower images were taken 5 s (PH–GFP) or 60 s (OSH2 and P4M) later. Tb^R332H^, OSH2, OSH1 and P4M were expressed under control of the Rh1 promoter, hence the central R7 rhbadomere appears as a dark hole lacking GFP; PH–GFP was expressed using the *trp* promoter and is also expressed in R7. (C) Representative PMT measurements of DPP fluorescence (sampled at 50 Hz from cropped regions, corresponding approximately to fields shown in A and B). Each probe decayed (translocated) with a characteristic timecourse similar to that seen in dissociated ommatidia (compare with Fig. 1B). No translocation was detected in the null PLC mutant (*norpA^P24^*). (D) Rapid changes in Tb^R332H^ fluorescence (sampled at 500 Hz). An initial rapid transient (arrow, the inset shows a faster timebase) reflects M to R photoisomerisation by the blue excitation and was not seen if M was not first reconverted into R by orange–red light (lower traces). (E) Rapid changes in PH–GFP fluorescence. After the R-to-M transient (arrow), fluorescence increased briefly (∼200 ms) before decaying. (F) Traces of fluorescence decay of P4M and Tb^R332H^ on wild-type (same traces as in C, but note the different timescale) and *trp* mutant backgrounds. (G) Time constants (tau, single exponential fits) of fluorescent decay traces in wild-type and *trp* backgrounds (mean±s.e.m., *n*=6–13). Decay was significantly (**P<0.005, two-tailed unpaired Student's *t*-test) faster in *trp* mutants for OSH2, P4M and Tb^R332H^, but slower than for PH–GFP. Decay for Tb^R332H^ was faster still (0.87 s) on the *trpl*;*trp* background. (H) Extent of translocation (*F*_max_*/F*_min_ ratio) for each probe was similar on wild-type and *trp* backgrounds (mean±s.e.m., *n*=5–12). Note that comparisons of *F*_max_*/F*_min_ between probes are not reliable because each transgene confers a different eye colour, which influences *F*_max_*/F*_min_ (see Materials and Methods and Fig. S2). PH–GFP was expressed on a *cn*, *bw* (white-eyed) background (hence low *F*_max_*/F*_min_ values); eye colour of others were various shades of orange.

Although fluorescent probes can be tracked with high resolution in dissociated ommatidia, this preparation is relatively short-lived (∼2 h) and vulnerable to damage by the intense illumination required to excite fluorescence. In order to obtain more quantitative data under physiological conditions, we exploited the optics of the compound eye to image fluorescence in the rhabdomeres in intact flies. As described in classic studies from the 1970s (e.g. Franceschini and Kirschfeld, 1971), by focussing a low-power objective below the surface of the eye where the optical axes of neighbouring ommatidia converge, the characteristic rhabdomere pattern is seen as a magnified virtual image known as the deep pseudopupil (DPP). Not only does this collect light from many tens of ommatidia, but because of their wave-guiding properties, the fluorescence of the rhabdomeres is directional and channelled to the objective, whereas fluorescence from the cell body is diffuse and comparatively weak (Fig. 2A). Fluorescence measured from the DPP thus provides a measure of the concentration of the probe in the rhabdomere. This non-invasive preparation is very robust, allowing repeated measurements over many hours, and provides a rare opportunity for live tracking of fluorescent probes in a subcellular compartment in completely intact animals (Meyer et al., 2006; Satoh et al., 2010).

Fig. 2 shows frames from movies (Movies 4 and 5) of the DPP from flies expressing four of the probes (Tb^R322H^, PH–GFP, OSH2 and P4M). At time zero (first frames) in dark-adapted flies, the initial rhabdomeric localisation is clearly visible, but within seconds the fluorescence from the rhabdomere pattern faded, often becoming darker than background fluorescence, reflecting translocation of the probe out of the rhabdomere due to depletion of PtdIns(4,5)*P*_2_ and PtdIns4*P* by light-activated PLC. The timecourses of fluorescence decay were very similar to those measured in dissociated cells expressing the respective probes (cf. Fig. 1C). Although it is straightforward to make measurements from ROIs of such movies, it is more convenient to directly measure intensity through a photomultiplier tube (PMT), using a rectangular diaphragm to crop the area of measurement to the rhabdomere pattern (e.g. Fig. 2). Timecourses derived from such measurements are similar to those made using ROI analysis of movies (e.g. Satoh et al., 2010), and allow measurements to be made in real time with high temporal resolution.

As well as confirming the decay of fluorescence, with distinct timecourses for the different probes, the PMT records also revealed additional rapid kinetic features. First, there was a very rapid transient with a decay time constant of ∼10–20 ms. This transient reflects R to M photoisomerisation and was absent in a second flash if the visual pigment was not first photoreisomerised to the R state by long-wavelength light (Fig. 2D). The rapid reduction in fluorescence is presumably because M (λ_max_ 570 nm) absorbs the emitted fluorescence more strongly than R (λ_max_ 480 nm). Interestingly, PH–GFP in particular then showed a distinct increase in fluorescence lasting ∼200 ms after light onset, before the decay reflecting PtdIns(4,5)*P*_2_ hydrolysis (Fig. 2E). Such a phase was generally absent using the other probes, and possibly reflects the initial rapid release of Ins*P*_3_ in the microvilli before it diffuses away into the cell body.

In order to confirm that the decay of the fluorescence was dependent upon hydrolysis of PtdIns(4,5)*P*_2_ by PLC, we crossed flies expressing PH–GFP and Tb^R332H^ into a PLC-null mutant background (*norpA^P24^*). As expected, apart from the rapid transient reflecting photoisomerisation of R into M, there was now essentially no loss of fluorescence over time (Fig. 2C). In principle, GFP fluorescence might be sensitive to other light-induced changes in the microvillar environment, such as pH, which can be expected to become more acidic during illumination because of the protons released by the PLC reaction (Huang et al., 2010). However, similar measurements from flies expressing other GFP-tagged proteins in the microvilli (including TRPL, INAD and Kir2.1) showed little or no dynamic signal (<5%) apart from the R or M transient (data not shown).

### Intensity dependence of phosphoinositide depletion

**Fig. 3.**
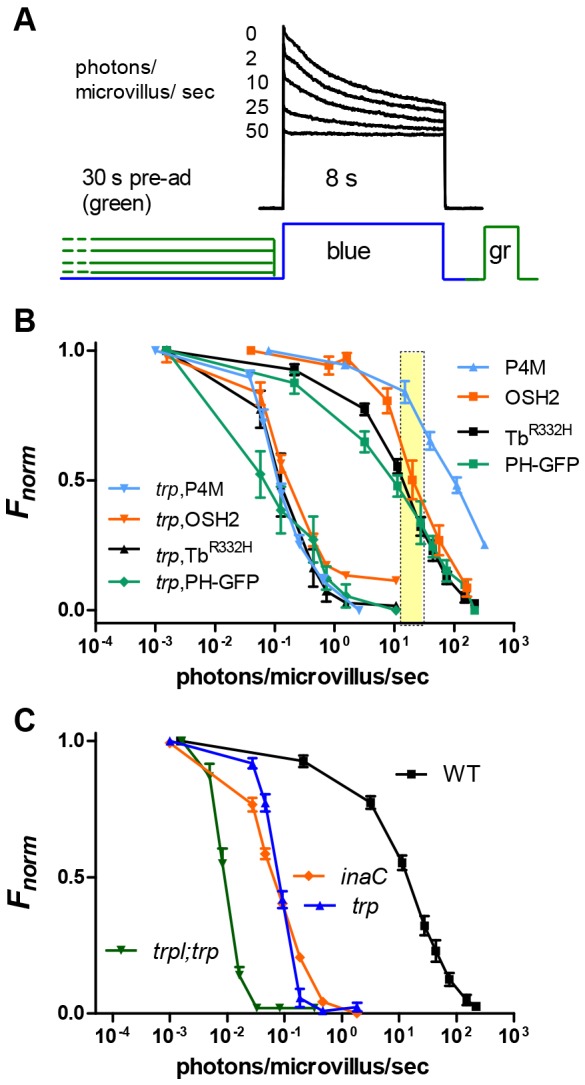
**Intensity dependence of phosphoinositide depletion.** (A) Tb^R332H^ fluorescence from the DPP of an intact fly. The fly was pre-adapted with varying intensities of long-wavelength (green, 545 nm bandpass) light for 30 s before measuring instantaneous fluorescence with blue excitation. M was then reconverted into R by photoequilibrating green (gr) illumination, and the fly dark-adapted for 2 min before the next measurement. (B) Fluorescence (normalised between *F*_max_ and *F*_min_) as a function of intensity of the pre-adapting light expressed in effectively absorbed photons/microvillus/s. On the wild-type background, the PtdIns4*P*-binding probes (OSH2 and P4M) required up to ∼10× brighter illumination for effective translocation. PH–GFP showed substantial depletion at slightly lower intensities than Tb^R332H^. In *trp* mutants, PtdIns(4,5)*P*_2_ and PtdIns4*P* were depleted with ∼100× dimmer illumination. Results are mean±s.e.m., *n*=6–10. The yellow bar represents estimate of brightest natural light levels (∼0.5×10^6^–1×10^6^ photons/photoreceptor/s). (C) Tb^332H^ fluorescence as function of pre-adapting intensity in wild type (replotted from B), *trp^343^* (*n*=15; independent data set from panel B), *inaC^P209^* (*n*=21) and *trpl*;*trp* (*n*=9). Results plotted as mean±s.e.m.

These results indicate that PtdIns(4,5)*P*_2_ and PtdIns4*P* are substantially depleted in wild-type flies by saturating blue illumination. In order to measure phosphoinositide turnover in response to more physiological light regimes, we used a three-pulse paradigm (Fig. 3). Flies were first exposed to a photoequilibrating 2–4 s flash of green illumination (545 nm, bandwidth 100 nm) that leaves most (>95%) of the visual pigment in the R state, and were then dark-adapted for 2 min. The flies were then pre-adapted for 30 s with different intensities of the same green light and the instantaneous fluorescence in the DPP measured with blue excitation after a negligible delay (200 ms). M was then again reconverted into R by photoequilibrating green light and the cycle repeated, each time varying the intensity of the pre-adaptation, which was calibrated in terms of the effectively absorbed photons (see Materials and Methods and Fig. S4). Data were normalised between *F*_max_ (fluorescence intensity after 2 min dark-adaptation, set at 1.0) and *F*_min_ (intensity after prolonged exposure to saturating blue excitation, set as 0). The relative amount of PtdIns*P*_n_ species remaining the in the rhabdomere after maximum depletion (i.e. corresponding to *F*_min_) is not known with certainty; however, there was unlikely to be much residual PtdIns(4,5)*P*_2_ because the DPP images (e.g. Fig. 2A) show that fluorescence of Tb^R332H^ dropped to levels below background and similar to levels in the central R7 rhabdomere which, because of the cell-specific promoter used (Rh1) used, did not express any probe. This would also accord with previous measurements using the PtdIns(4,5)*P*_2_-sensitive Kir2.1 channel in which channel activity was suppressed to <5–10% of control levels by PtdIns(4,5)*P*_2_-depleting stimuli (Hardie et al., 2004). By contrast, DPP images of P4M generally revealed a substantial residual fluorescence above background and R7 suggesting that PtdIns4*P* might not have been quite so severely depleted (Fig. 2B).

All probes behaved in a broadly similar fashion, translocating from the rhabdomere progressively with increasing pre-adaptation intensity (Fig. 3B). Tb^R332H^ and PH–GFP showed 50% depletion at intensities equivalent to ∼10 effectively absorbed photons/microvillus/s (or ∼300,000 photons per second per photoreceptor), approximating bright daylight conditions (Juusola and Hardie, 2001). PH–GFP began to leave the rhabdomeres at slightly lower intensities than Tb^R332H^. Again this might reflect the high affinity of PH–GFP for Ins*P*_3_. Thus, even before substantial depletion of PtdIns(4,5)*P*_2_, finite amounts of InsP_3_ are generated in the rhabdomeres, and will presumably diffuse into the cell body, where it would act as a high-affinity sink for PH–GFP.

OSH2, which binds PtdIns4*P* and PtdIns(4,5)*P*_2_ with similar affinity, was somewhat more resistant to depletion, but the PtdIns4*P*-specific P4M probe was most resistant, with only partial depletion observed at the highest green intensities tested, which were at least an order of magnitude brighter than brightest environmental light levels. Taken together, these results indicate that a substantial (≥50%) reserve of PtdIns(4,5)*P*_2_ and most of the PtdIns4*P* remain in the microvilli of wild-type flies even under the brightest natural conditions.

### Phosphoinositide depletion in *trp* and PKC mutants

The transient receptor potential (*trp*) mutant is so-called because its response to maintained illumination decays to baseline over a few seconds (e.g. Fig. 7B). From experiments using Kir2.1 channels as biosensors, we have previously proposed that the *trp* decay phenotype reflected depletion of PtdIns(4,5)*P*_2_ due to failure of Ca^2+^-dependent inhibition of PLC by Ca^2+^ influx through the TRP channels (Hardie et al., 2001). To test this *in vivo* with independent methodology, we expressed the probes in *trp* mutants and again quantified their time- and intensity-dependent translocation.

Consistent with enhanced PtdIns(4,5)*P*_2_ hydrolysis in *trp* mutants, Tb^R332H^, OSH2 and P4M all translocated significantly more rapidly (*P*<0.005, two-tailed unpaired Student's *t*-test) in *trp* mutant backgrounds (Fig. 2G). Interestingly, however, PH–GFP translocation was actually slightly slower. This might again reflect its affinity for InsP_3_, which should continue to be produced within the microvilli for longer if PLC is not rapidly inactivated. Most strikingly, when we determined the intensity dependence of depletion, all probes translocated out of the rhabdomeres at ∼100 times lower intensities in *trp* than in wild-type flies (Fig. 3B), confirming that there is a profound PtdIns(4,5)*P*_2_ depletion as has previously been proposed to underlie the *trp* decay phenotype (Hardie et al., 2004, 2001), and extending this finding now to PtdIns4*P*. Nevertheless, the overall extent of translocation (*F*_max_/*F*_min_ ratio) following prolonged (≥30 s) blue excitation was similar in wild-type and *trp* backgrounds for both the PtdIns(4,5)*P*_2_- and the PtdIns4*P*-binding probes (Fig. 2H). This indicates that, in the PDA state (after photoequilibrating blue illumination), PtdIns4*P* and PtdIns(4,5)*P*_2_ are depleted in wild-type flies to a similar extent to that observed in *trp* mutants.

The depletion of PtdIns(4,5)*P*_2_ in *trp* mutants has been attributed to the failure of Ca^2+^-dependent inhibition of PLC, but how Ca^2+^ inhibits PLC is unclear. Previously we proposed that the inhibition was mediated by protein kinase C (PKC), encoded by the *inaC* gene (Gu et al., 2005). If this is the case, we predicted that in *inaC* mutants, PtdIns(4,5)*P*_2_ should also be depleted at much lower intensities than in wild-type flies. Indeed, PtdIns(4,5)*P*_2_ depletion monitored using Tb^R332H^ in the PKC-null mutant *inaC^P209^* was at least as severe as in the *trp* mutant, even showing some loss of probe from the rhabdomere pattern in the DPP at intensities below the threshold for depletion in a *trp* background (Fig. 3C).

In *trp* mutants, the remaining TRPL channels still permeate substantial amounts of Ca^2+^ before the response decays to baseline. To investigate PtdIns(4,5)*P*_2_ depletion in the absence of any Ca^2+^ influx, we expressed Tb^R332H^ in a *trpl*;*trp* double mutant background lacking all light-sensitive channels. PtdIns(4,5)*P*_2_ was now depleted even more sensitively (∼10-fold) than in single *trp* or *inaC* mutants (Fig. 3C). The rate of Tb^R332H^ translocation on a *trpl*; *trp* background was also very significantly accelerated with respect to the single *trp* mutant, with a time constant of <1 s (0.87±0.46 s, mean±s.e.m., *n*=7; Fig. 2G).This suggests that in addition to inhibition mediated by PKC, there is also a PKC-independent and Ca^2+^-dependent inhibition of PLC.

### Phosphoinositide resynthesis

**Fig. 4.**
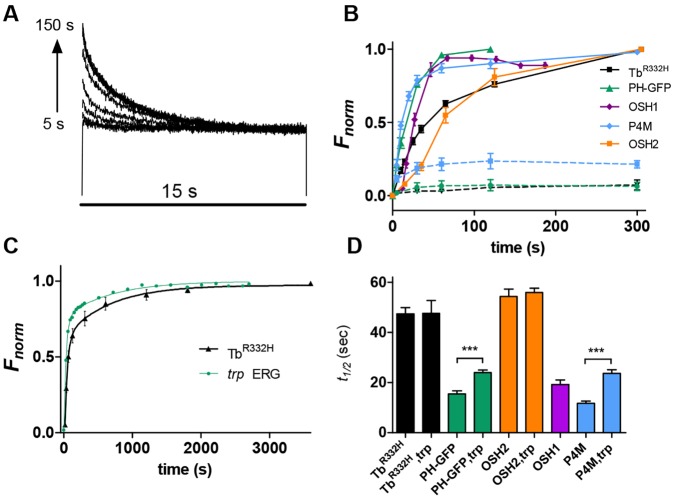
**Timecourse of phosphoinositide resynthesis.** (A) Superimposed series of fluorescence traces measured from the DPP of a fly expressing Tb^R332H^. Before each trace, M was reconverted into R with photoequilibrating long-wavelength illumination and then left in the dark for varying durations (5–150 s). Fluorescence recovered progressively, reflecting return of the PtdIns(4,5)*P*_2_-specific Tb^R332H^ probe to the rhabdomeres and then decayed again during the 15 s blue excitation light. (B) Averaged, normalised (between *F*_max_ and *F*_min_) recovery timecourses for five different probes (mean±s.e.m., *n*=6–12 flies). Dotted lines, Tb^R332H^, PH–GFP and P4M measured without first photoreisomerising M into R showed little or no recovery. (C) On a longer time scale, Tb^R332H^ showed a slow component of recovery taking up to ∼1 h to fully recover; a similar slow component was seen in the recovery of the ERG response in *trp*. Data fitted with two exponential fits (Tb^R332H^ 38 s and 642 s; *trp* ERG 35 s and 776 s). Results are mean±s.e.m., *n*=4. (D) Half times (*t*_1/2_) for recovery of fluorescence (to 50% of value after 5 min of dark adaptation) were substaintially slower on *trp* backgrounds for PH–GFP and P4M (***P<0.001, two-tailed unpaired Student's *t*-test), but not for Tb^R332H^ or OSH2. Results are mean±s.e.m. (*n*=7–10 flies).

Previous measurements using Kir2.1 channels as electrophysiological biosensors indicated that, following depletion, PtdIns(4,5)*P*_2_ is resynthesised over a timecourse of a couple of minutes in whole-cell voltage-clamped photoreceptors (Hardie et al., 2004). We tracked phosphoinositide resynthesis *in vivo* in intact flies by measuring the timecourse with which fluorescence returned to the rhabdomeres following depletion. Flies were first exposed to 30–60 s blue illumination, resulting in maximum loss of probe from the DPP; M was then photoreisomerised to R with a 2–4-s photoequilibrating flash of long-wavelength light and, after a variable period of dark adaptation, fluorescence in the DPP was measured with another blue stimulus (Fig. 4). The cycle was repeated, each time varying the time in the dark, until the most of the fluorescence in the rhabdomeres had recovered.

#### PtdIns(4,5)*P*_2_ probes

The PtdIns(4,5)*P*_2_- and InsP_3_-binding PH–GFP returned very quickly with a half-time (*t*½) of only 15 s. Tb^R332H^ returned with a *t*½ of ∼40 s, which is close to that reported using Kir2.1^R228Q^ (∼50 s) in whole-cell recordings (Hardie et al., 2004). Tb^R332H^ returned with a similar timecourse on both wild-type and *trp* mutant backgrounds (Fig. 4D); however, PH–GFP returned significantly more slowly on a *trp* mutant background (*t*½ ∼25 compared with 15 s in wild type), possibly reflecting slow cytosolic clearance of the excess InsP_3_ generated in *trp* mutants (because of prolonged PLC activity). On a longer time scale, Tb^R332H^ returned with two distinct time constants, reaching ∼75% recovery in 1–2 min, but requiring ∼1 h for complete recovery (Fig. 4C). We looked for a functional correlate of this in the recovery of light sensitivity in electroretinogram (ERG) recordings from *trp* mutants after inducing response decay with prolonged (20 s) saturating illumination. Indeed, we found that whereas most sensitivity to light recovered relatively rapidly (∼80% within ∼2 min), there was a second slower phase of recovery with sensitivity continuing to increase for up to 1 h after the initial PtdIns(4,5)*P*_2_ depletion (Fig. 4C). A two-exponential fit to the data yielded very similar time constants (35 s and 776 s) to those seen using Tb^R332H^ (38 s and 642 s).

Whether measured in wild-type or *trp* mutants, the timecourse for PtdIns(4,5)*P*_2_ resynthesis in the dark seemed quite slow compared to the rate of depletion, and we wondered whether resynthesis might be accelerated during illumination by Ca^2+^ or other products of the transduction cascade. To test this, we measured recovery timecourses using Tb^R332H^ (in a wild-type background), both in the dark and in the presence of background intensities sufficient to generate a sizeable electrical light response (with associated Ca^2+^ influx). Over a ∼100-fold range of intensities (∼0.1–10 photons/microvillus/s), from those too low to induce any PtdIns(4,5)*P*_2_ depletion to those inducing >50% PtdIns(4,5)*P*_2_ depletion, we found no indication for an acceleration of resynthesis, suggesting the rate-limiting step(s) for PtdIns(4,5)*P*_2_ resynthesis are not modulated by Ca^2+^ or other products of the phototransduction cascade (Fig. S3).

#### PtdIns4*P* probes

Of the three PtdIns4*P* probes tested, OSH1 and P4M both recovered quickly, with *t*½ of 26 s (OSH1) and 12 s (P4M) respectively. By contrast, OSH2 returned much more slowly (*t*½ 55 s). OSH2 has the highest affinity of these probes (Yu et al., 2004), and also exhibited the slowest rate of translocation from rhabdomere to cytosol (Fig. 2), suggesting that its translocation might be compromised by high-affinity or non-specific binding. OSH1 had relatively rapid recovery kinetics; however, because, like OSH2, it is compromised by also binding to PtdIns(4,5)*P*_2_ and also gave a weak *F*_rh_/_cyt_ ratio (Fig. 1), we consider the PtdIns4*P-*specific P4M probe to be the most reliable reporter.

P4M behaved in a broadly similar manner when expressed in *trp* mutants; however, recovery was ∼2-fold slower (*t*½ 23 s). The slower recovery might reflect more profound phosphatidylinositol depletion during blue illumination in *trp* mutants necessitating replenishment from the endoplasmic reticulum (ER), whereas PtdIns4*P* in wild type might be at least partially restored from a phosphatidylinositol reserve remaining in the rhabdomere.

### PtdIns(4,5)*P*_2_ recycling mutants

**Fig. 5.**
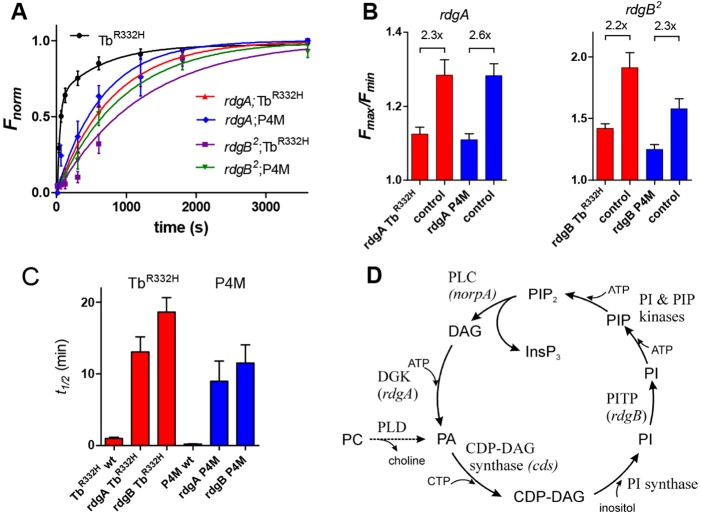
**Phosphoinositide resynthesis in *rdgB* and *rdgA* mutants.** (A) Normalised recovery of DPP fluorescence in flies expressing P4M and Tb^R332H^ following PtdIns(4,5)*P*_2_ depletion in wild-type (Tb^R332H^ only, replotted from Fig. 4), *rdgB^2^* and *rdgA^1^*;;*trp^343^* mutant backgrounds. PtdIns(4,5)*P*_2_ and PtdIns4*P* resynthesis were greatly slowed in both *rdgA* and *rdgB* mutants, but still recovered over ∼1 h. Results are mean±s.e.m., *n*=4–6. Data fitted with exponentials (*rdgA*;*Tb* 805 s; *rdgA*;*P4M* 650 s, *rdgB;P4M* 950 s; *rdgB*;*Tb* 1250 s). (B) *F*_max_*/F*_min_ ratios in *rdgA^1^*;;*trp* and *rdgB^2^* compared to eye-colour-matched wild-type or *trp* controls expressing the same transgenes. The fold-reduction in *F*_max_*/F*_min_ (indicated above each pair) provides an estimate of the reduction in maximum (dark-adapted) PtdIns(4,5)*P*_2_ and PtdIns4*P* levels. Results are mean±s.e.m. (*n*=4–8). (C) Half times (*t*½) for recovery of Tb^R332H^ and P4M in *rdgA^1^*;;*trp^343^* and *rdgB^2^* and wild-type controls (mean±s.e.m., *n*=4–8). (D) Proposed phosphoinositide cycle in *Drosophila* photoreceptors. Note that phosphatidic acid (PA) could in principle also be generated from phosphatidyl choline (PC) by phospholipase D (PLD).

The first enzyme in the canonical PtdIns(4,5)*P*_2_ recycling pathway is a DAG kinase (DGK), which converts DAG into phosphatidic acid (Fig. 5D). The DGK in *Drosophila* photoreceptors is encoded by the *rdgA* gene (Masai et al., 1993); however, there is little direct evidence that it is required for PtdIns(4,5)*P*_2_ resynthesis in the photoreceptors, and in principle there are alternative sources of phosphatidic acid, such as from phosphatidyl choline through the action of phospholipase D (e.g. Raghu et al., 2009). Because severe *rdgA* mutants have profound early onset retinal degeneration due to constitutive Ca^2+^ influx through TRP channels, we investigated PtdIns(4,5)*P*_2_ resynthesis in *rdgA*;;*trp* double mutants, in which degeneration is largely rescued (Raghu et al., 2000). When expressed in dark-adapted *rdgA^1^*;;*trp^343^* double mutants, we detected fluorescence of Tb^R332H^ in the rhabdomeres, which decayed as usual during blue illumination. However, subsequent return of Tb^R332H^ in the dark (following photoreisomerisation of M to R) was greatly slowed (*t*½ ∼13±5.5 min, mean±s.e.m., *n*=7; Fig. 5). Given that *rdgA^1^* is not a null allele, we cannot conclude whether the eventual recovery of PtdIns(4,5)*P*_2_ was due to residual DGK function or whether alternative, slower pathways were responsible. Both absolute fluorescence intensities and *F*_max_/*F*_min_ ratios in *rdgA*;;*trp* were also reduced. On the assumption that PtdIns(4,5)*P*_2_ was ultimately depleted to at least the same extent by blue excitation in *rdgA*;;*trp*, comparison of *F*_max_/*F*_min_ values with controls should provide information on the initial dark-adapted level of PtdIns(4,5)*P*_2_*.* This comparison suggests that resting PtdIns(4,5)*P*_2_ is at least ∼2-fold reduced compared to controls (Fig. 5B).

Another gene presumed to be required for PtdIns(4,5)*P*_2_ recycling is *rdgB*, which encodes a phosphatidyl inositol transfer protein (PITP) believed to be required for transporting phosphatidylinositol from the ER to the plasma membrane (Milligan et al., 1997; Vihtelic et al., 1993). Recently RDGB and a mammalian homologue (Nir2, also known as PITPNM1) have also been proposed to transport phosphatidic acid in the opposite direction (Kim et al., 2015; Yadav et al., 2015). Mutants of *rdgB* undergo severe light-dependent retinal degeneration, but have wild-type morphology when reared in the dark. *rdgB* mutations have also been shown to result in prolonged response inactivation (Milligan et al., 1997) and to impair resynthesis of PtdIns(4,5)*P*_2_ in dissociated cells in experiments using Kir2.1 channels as a biosensor (Hardie et al., 2001). When Tb^R332H^ was expressed in a protein-null *rdgB* mutant (*rdgB^2^*), the rhabdomere pattern in the DPP of dark-reared flies again showed clear fluorescence, which decayed during blue excitation. However, recovery of fluorescence was profoundly slowed, recovering over a period of ∼1 h with a timecourse (*t*½ 18.6±5.9 min, *n*=8) even slower than that observed in *rdgA* mutants (Fig. 5C). Based on the *F*_max_/*F*_min_ ratios, the fully dark-adapted level of PtdIns(4,5)*P*_2_ in *rdgB^2^* was ∼2.2-fold reduced compared to controls (Fig. 5B). Experiments using the hypomorphic allele *rdgB^9^* indicated a similar, but slightly less severe slowing of PtdIns(4,5)*P*_2_ resynthesis (*t*½ 13.1±2.0 min, mean±s.e.m., *n*=9) and reduction in *F*_max_/*F*_min_ values (2.1-fold).

We also confirmed that both DGK and the PITP protein RDGB were required for replenishing PtdIns4*P* in the rhabdomere by expressing P4M in *rdgA^1^* and *rdgB^2^* backgrounds. Following depletion, fluorescence in both *rdgA^1^*;;*trp,*P4M and *rdgB^2^*;P4M flies again recovered only slowly over a period of ∼1 h (*t*½ ∼9 min in *rdgA^1^*;;*trp* and ∼12 min in *rdgB^2^*). Based on the *F*_max_/*F*_min_ ratios, the fully dark-adapted level of PtdIns4*P* in both *rdgA^1^*;;*trp* and *rdgB^2^* was also substantially (∼2.5-fold) reduced (Fig. 5B).

### Simultaneous fluorescence and ERG recordings

**Fig. 6.**
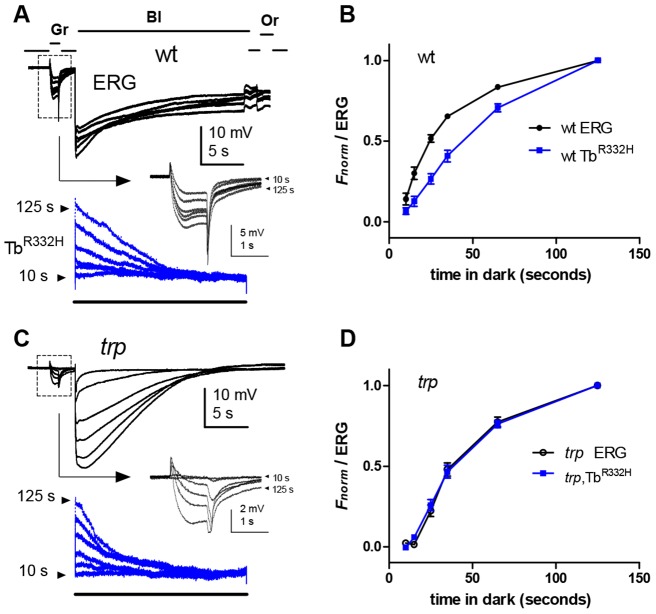
**Simultaneous recordings of recovery from Tb^R332H^ and ERG.** (A) A 1-s green (Gr) test flash to test sensitivity in the ERG (top traces) was delivered prior to blue (Bl) illumination for 20 s to excite Tb^R332H^ fluorescence (lower, blue traces). The arrow points to responses to the test flash (boxed) on an expanded scale. Finally, an intense orange (Or) flash was delivered to reconvert M into R. The period in the dark between each sequence was varied from 10–120 s. In both wild-type (A,B) and *trp* (C,D) backgrounds, PtdIns(4,5)*P*_2_ (Tb^R332H^ signal) was depleted by the blue excitation and resynthesised with a similar time course. (B) On the wild-type background, the normalised ERG response amplitude to the green test flash recovered substantially more quickly than Tb^R332H^ fluorescence (normalised between *F*_max_ and *F*_min_); (D) whereas on the *trp* background the normalised ERG recovered with an almost identical timecourse. Plots in B and D are normalised mean data±s.e.m., *n*=10 (wild-type) or 7 (*trp*).

**Fig. 7.**
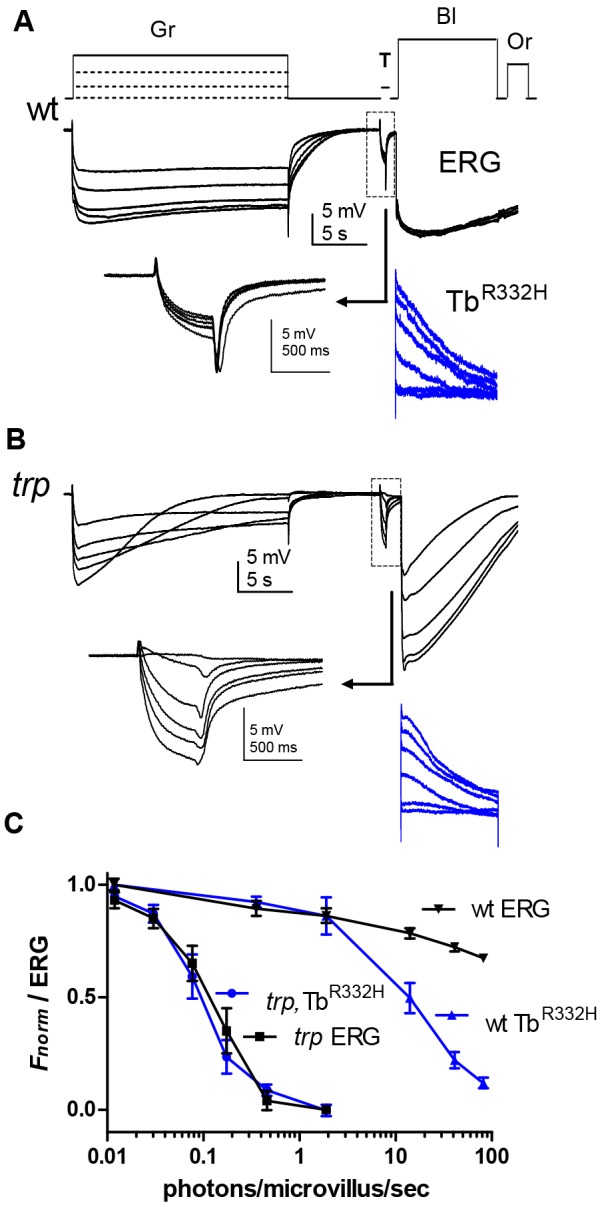
**Simultaneous ERG and Tb^R332H^ measurements of the intensity dependence of PtdIns(4,5)*P*_2_ depletion.** (A) An intact wild-type fly expressing Tb^R332H^ was first exposed to a 25-s pre-adapting green (Gr) illumination of varying intensities, and sensitivity tested with a brief (500 ms) test flash (T, boxed region; arrow points to the test response on an expanded scale) immediately before measuring instantaneous fluorescence with blue (Bl) excitation (Tb^R332H^, blue traces). Orange (Or) light then reconverted M into R, and the flies were dark-adapted for 2 min before repeating with the next pre-adapting intensity. (B) Similar protocol in a *trp* mutant, using ∼100× dimmer pre-adapting regimes (note *trp* decay at higher intensities). (C) Averaged normalised data (mean±s.e.m., *n*=7) comparing an ERG test flash and Tb^R332H^ fluorescence as a function of pre-adapting intensity. In *trp* mutants, Tb^R332H^ and ERG data essentially overlap. On the wild-type background, ERG sensitivity was only slightly reduced even after the brightest pre-adapting light (equivalent to ∼100 effective photons/microvillus/s), which had depleted ∼90% of the PtdIns(4,5)*P*_2_.

Both the intensity dependence of PtdIns(4,5)*P*_2_ depletion and the timecourse of its recovery appear similar to measurements previously made of sensitivity to light in *trp* mutants using electrophysiological recordings (Cosens and Manning, 1969; Hardie et al., 2004, 2001; Minke, 1982). However, these were made using different experimental conditions and illumination regimes. In order to test this relationship more rigorously, we made simultaneous recordings of ERG and Tb^R332H^ fluorescence, so that PtdIns(4,5)*P*_2_ levels could be directly compared with sensitivity to light in the same fly. Strikingly, sensitivity to light in *trp* mutants was suppressed by pre-illumination with essentially identical intensity dependence to that of the PtdIns(4,5)*P*_2_ depletion monitored by Tb^R332H^, and recovered with an almost identical timecourse (Figs 6B and 7B). These results represent compelling quantitative confirmation that the *trp* phenotype reflects PtdIns(4,5)*P*_2_ depletion and indicate that the response to light in *trp* (mediated by TRPL channels) can also be used as a sensitive indicator of PtdIns(4,5)*P*_2_ levels.

We also made simultaneous recordings of ERG and fluorescence in wild-type flies, where the response is predominantly mediated by TRP channels (Figs 6A and 7A). Although Tb^R332H^ returned to the rhabdomere with a similar timecourse to that observed in *trp* mutants, sensitivity to light recovered substantially more quickly. Furthermore, suppression of sensitivity to light by pre-adapting illumination was much less than the extent of PtdIns(4,5)*P*_2_ depletion [e.g. ∼70% ERG amplitude remaining after PtdIns(4,5)*P*_2_ had been depleted to 10% of original level]. This distinction indicates that TRP channels can still be strongly activated when PtdIns(4,5)*P*_2_ levels are much lower than is the case for TRPL channels, and is likely to be a contributory factor to the ‘transient receptor potential’ response decay phenotype whereby the response decays to baseline as PtdIns(4,5)*P*_2_ is depleted due to failure of Ca^2+^-dependent inhibition of PLC

### Timecourse of synthesis of PtdIns(4,5)*P*_2_ from PtdIns4*P*

Somewhat surprisingly, recovery of Tb^R332H^ (*t*½ ∼40 s) was much slower than for P4M (∼12 s), suggesting that conversion of PtIns4*P* into PtdIns(4,5)*P*_2_ by PtdIns4*P* 5-kinase is the slowest, and rate-limiting, step in the phosphoinositide cycle of the photoreceptors (Fig. 4). In order to obtain an independent estimate of this rate, we expressed the *Ciona intestinalis* voltage-sensitive lipid phosphatase (Ci-VSP) in the photoreceptors. This unique voltage-dependent enzyme rapidly dephosphorylates PtdIns(4,5)*P*_2_ to PtdIns4*P* at positive membrane voltages, providing an opportunity to measure how fast PtdIns(4,5)*P*_2_ is resynthesised from PtdIns4*P* (Falkenburger et al., 2010; Iwasaki et al., 2008; Murata et al., 2005). To exploit this tool, experiments must be performed under voltage clamp conditions, and rather than using lipid probes to monitor PtdIns(4,5)*P*_2_ levels, we now simply used the response to light mediated by TRPL channels, which, as shown above (Figs 6 and 7), can also provide a valid measure of PtdIns(4,5)*P*_2_.

**Fig. 8.**
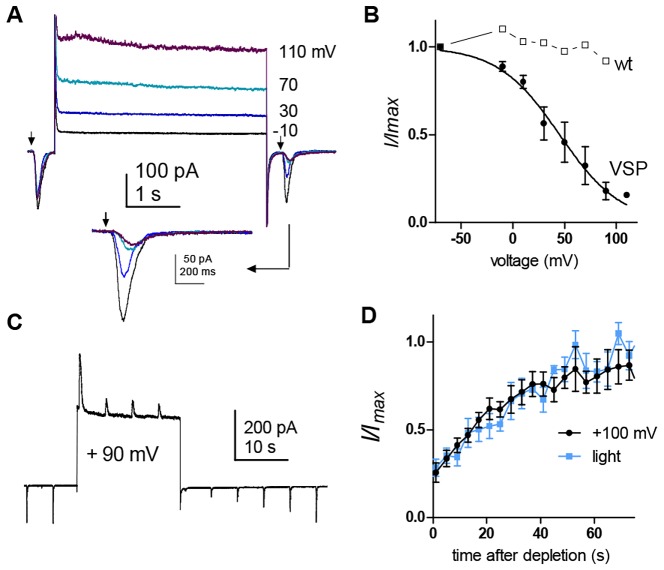
**Depletion of PtdIns(4,5)*P*_2_ by *Ciona* voltage-sensitive phosphatase.** (A) Whole-cell recording from photoreceptor expressing Ci-VSP in presence of 100 µM La^3+^ to isolate the TRPL current. Following a brief test flash (arrow), 4-s voltage steps (−10 to +110 mV from a holding potential of −70 mV) were applied and sensitivity retested with a second test flash immediately on return to −70 mV (inset below on expanded scale). (B) Response of second test flash normalised to control prior to the voltage step (*I*/*I*_max_) as a function of voltage. Data fitted with a Boltzmann distribution with *V*_50_ +45 mV (mean±s.e.m., *n*=4). Similar data from a control wild-type cell (wt) showed no suppression. (C) Continuous recording showing responses to light flashes suppressed during a +90 mV voltage step (currents now outward), recovering over a period of ∼30 s on return to −70 mV. (D) Averaged recovery time course data (normalised to test flash prior to voltage step ending at time zero; mean±s.e.m., *n*=7), compared to the recovery timecourse of the light response following PtdIns(4,5)*P*_2_ depletion by light stimuli calibrated to result in the same initial suppression in the same cells.

Photoreceptors from dissociated ommatidia expressing Ci-VSP were whole-cell voltage-clamped, replacing K^+^ with Cs^+^ and tetraethylammonium (TEA^+^) in the pipette solution to block voltage-dependent K^+^ channels, and exposed to La^3+^ to block TRP channels, leaving a light-induced current mediated exclusively by TRPL channels (phenocopying the *trp* mutation). After recording control responses to dim test flashes at resting potential (−70 mV), voltage steps were applied between −10 and +110 mV and the response to the same test flash recorded on return to −70 mV (Fig. 8A,B). The response to the second flash was systematically suppressed as a function of the voltage, closely matching the known voltage dependence of Ci-VSP (Falkenburger et al., 2010; Murata and Okamura, 2007). Controls in wild-type photoreceptors showed no such suppression by depolarising steps. The suppression of sensitivity to light can be attributed to Ci-VSP activity depleting PtdIns(4,5)*P*_2_, and the rate of PtdIns(4,5)*P*_2_ resynthesis from PtdIns4*P* can thus be estimated from the timecourse with which sensitivity to light recovered. Responses following a near-saturating voltage step (5–10 s to +90 mV) were suppressed to ∼20% of their initial value and recovered, typically to within ∼80% of control, over a period of 1–2 min (Fig. 8C). We then depleted PtdIns(4,5)*P*_2_ to the same extent in the same cell, by using light-adjusted in intensity, in order to induce a similar initial suppression. The recovery timecourse from the light-induced and Ci-VSP-induced suppression overlapped closely, supporting the idea that conversion of PtIns4*P* into PtdIns(4,5)*P*_2_ by PtdIns4*P* 5-kinase indeed represents the rate-limiting step (Fig. 8D).

## DISCUSSION

Since the introduction of PLCδ1-PH–GFP (Stauffer et al., 1998; Varnai and Balla, 1998), fluorescently tagged lipid-binding domains have been widely used to monitor phosphoinositide turnover (reviewed in Balla et al., 2009), but in most cases their use has been restricted to cultured cells. In the present study, we systematically and quantitatively studied the translocation of a palette of PtdIns(4,5)*P*_2_- and PtdIns4*P*-specific probes *in vivo* in *Drosophila* photoreceptors in response to accurately calibrated stimulation. The DPP in particular provides a rare opportunity to track probes in a subcellular compartment (rhabdomere) in completely intact animals over extended time periods. As well as independently confirming conclusions from previous studies using Kir2.1 channels as electrophysiological biosensors (Hardie et al., 2004, 2001), the present study has provided substantial new insight into the dynamics of phosphoinositide turnover *in vivo*. The optical *in vivo* recordings are straightforward to perform, require only modest equipment and could well be used for relatively high-throughput screening. In the following sections, we discuss the relative merits of the different probes and the implications of the results for understanding phosphoinositide turnover *in vivo* under defined physiological conditions in this genetically tractable model system.

### PtdIns(4,5)*P*_2_ probes

The Tubby PH domain binds PtdIns(4,5)*P*_2_, PtdIns(3,4)*P*_2_ and PtdIns(3,4,5)*P*_3_, but not PtdIns(3,5)*P*_2_, PtdIns(4)*P*, PtdIns or Ins*P*_3_ (Brown et al., 2007; Santagata et al., 2001). Compared to PtdIns(4,5)*P*_2_, only trace amounts of PtdIns(3,4)*P*_2_, and PtdIns(3,4,5)*P*_3_ are likely to be present in the plasma (microvillar) membrane, and hence Tubby should effectively function as a specific PtdIns(4,5)*P*_2_ reporter. However, as noted in other systems (e.g. Quinn et al., 2008; Szentpetery et al., 2009), the affinity of wild-type Tubby protein appears to be too high to allow rapid dissociation from the membrane, and in photoreceptors it translocated too slowly to be useful. By contrast, the Tb^R332H^ mutant appeared to faithfully report PtdIns(4,5)*P*_2_ levels, and its behaviour – both intensity dependence of translocation and the timecourse of recovery – closely matched previous data obtained from whole-cell patch clamp using the Kir2.1^R228H^ mutant, which was similarly mutated to reduce effective affinity to match the physiological range (Hardie et al., 2004), as well as sensitivity to light in the *trp* mutant (Figs 6 and 7).

The widely used PLCδ1 PH domain (PH–GFP) gave broadly similar results to Tb^R332H^, but we noted several discrepancies. These included greater sensitivity to translocation at lower light levels, the very rapid time constant of translocation out of the rhabdomeres, an unusually rapid return to the rhabdomeres, a transient increase at the onset of blue excitation and paradoxically slower translocation out of the rhabdomeres in *trp* mutants, despite the expectation of more rapid PtdIns(4,5)*P*_2_ depletion. As discussed above, most of these differences seem attributable to the affinity of PH–GFP for InsP_3_ (Hirose et al., 1999; Nash et al., 2001; Szentpetery et al., 2009); however, the more rapid recovery following PtdIns(4,5)*P*_2_ depletion might imply that PH–GFP has a higher affinity for PtdIns(4,5)*P*_2_ than Tb^R332H^, although to our knowledge there are no direct data on their relative affinities. Although a useful probe for PLC activity, we therefore suggest that PLCδ1-PH should be used with caution as a reporter of PtdIns(4,5)*P*_2_ levels, in this system at least.

The very rapid translocation of PH–GFP with a time constant of ∼1 s (Fig. 1) is probably strongly influenced by the generation of InsP_3_ in the microvilli diffusing rapidly into the cytosol, where it acts as a sink. An alternative suggestion would be that the slower translocation of Tb^R332H^ and other probes reflects slower inherent diffusion of these probes (e.g. because of higher affinity or non-specific binding). However, this seems unlikely because in *trpl*;*trp* mutants, where net PtdIns(4,5)*P*_2_ hydrolysis is maximised due to the lack of all Ca^2+^ influx (Fig. 3C), Tb^R332H^ translocated just as rapidly as PH–GFP (Fig. 2G). Nevertheless, even this rapid translocation might ultimately be limited by diffusion, as other estimates of PtdIns(4,5)*P*_2_ depletion rates in the absence of all Ca^2+^ influx have suggested even more rapid hydrolysis. These include the rate of Shab (Kv2.1) channel modulation by light-induced PtdIns(4,5)*P*_2_ depletion (Krause et al., 2008), the rate of photomechanical contraction attributed to PtdIns(4,5)*P*_2_ depletion (Hardie and Franze, 2012) and the timecourse of acidification attributed to proton release by PLC (Huang et al., 2010). All these approaches suggest that PtdIns(4,5)*P*_2_ can be largely depleted within ∼200 ms by saturating light in the absence of Ca^2+^-dependent feedback.

During preparation of this manuscript, Chakrabarti et al. (2015) made measurements interpreted as PtdIns(4,5)*P*_2_ resynthesis, using PH–GFP imaged in the DPP, but reported ∼10-fold slower timecourses of recovery than those found here with PH–GFP (*t*½ 2–3 min compared with ∼15 s). A likely reason for this discrepancy is that they measured recovery of fluorescence (following blue excitation) in the presence of continuous red illumination, which was used to reconvert M into R. Because PtdIns(4,5)*P*_2_ remains depleted following blue excitation until M is photoreisomerised (Fig. 4B), it seems possible that the slow timecourses reported by these authors represented the time taken for M to R reconversion by their red illumination. By contrast we rapidly reconverted M into R with a brief (2–4 s) pulse of intense red–orange light before measuring the resynthesis timecourse in the dark.

### PtdIns4*P* probes

Of the three PtdIns4*P* probes tested, our results suggest that P4M is the most reliable. Until recently OSH1 and OSH2 were favoured probes for PtdIns4*P*; however, both also bind to PtdIns(4,5)*P*_2_ with similar affinity (Yu et al., 2004). By contrast the P4M domain (from *Legionella* SidM) is highly specific for PtdIns4*P* (Brombacher et al., 2009). Its affinity also appears well matched to the physiological levels of PtdIns4*P* in cultured cells because it responds to both increases and decreases in the abundance of the lipid (Hammond et al., 2014). Here, the close quantitative correspondence of intensity dependence of Tb^R332H^ and P4M translocation (Fig. 4) in *trp* mutants strongly suggests that the affinity of P4M is also well matched to the range of physiological levels of PtdIns4*P* in the photoreceptors.

The intensity dependence of OSH2 and OSH1 (not shown) translocation in wild-type photoreceptors was intermediate between the P4M and Tb^R332H^ probes (Fig. 3), consistent with their translocation being determined by a combination of PtdIns4*P* and PtdIns(4,5)*P*_2_ binding. OSH2 also displayed a very sluggish recovery timecourse and slow decay time constant suggesting that its translocation might be compromised by high-affinity or non-specific binding. Translocation of OSH1, which has ∼3-fold lower affinity than OSH2, might provide more plausible timecourse information, but its dual affinity for PtdIns4*P* and PtdIns(4,5)*P*_2_ as well as its relatively small *F*_max_*/F*_min_ signal (∼1.3 in dissociated ommatidia) compromise its usefulness.

### Phosphoinositide turnover under physiological conditions

The present results document the intensity dependence and resynthesis timecourse of both PtdIns(4,5)*P*_2_ and PtdIns4*P* under physiological conditions in wild-type backgrounds, and do so *in vivo* using completely intact living flies and illumination calibrated in terms of effectively absorbed photons. The results indicate that PtdIns(4,5)*P*_2_ can be depleted by only ∼50% at intensities expected to be experienced under brightest daylight conditions. Measurements using P4M indicated that PtdIns4*P* levels were virtually unaffected at physiologically relevant levels and only ∼50% depleted by intensities ∼10× brighter than daylight. However, an important finding was, that during the PDA caused by saturating blue illumination, both PtdIns(4,5)*P*_2_ and PtdIns4*P* appear to be depleted as extensively in wild-type as is the case in *trp* mutants and remained so until M had been reconverted into R by long-wavelength illumination. Although these conditions would rarely, if ever, occur under natural conditions, the PDA is extensively used in *Drosophila* eye research, and it is important to realise that in this state the microvilli are very likely severely depleted of both PtdIns(4,5)*P*_2_ and PtdIns4*P*.

### Mutations affecting the phosphoinositide cycle

The results provide compelling confirmation of the profound depletion of PtdIns(4,5)*P_2_* by low light levels in *trp* mutants, and now also extend this finding to PtdIns4*P* (Fig. 4). Quantitatively, ∼50% of both PtdIns(4,5)*P*_2_ and PtdIns4*P* (i.e. normalised probe fluorescence) were depleted in *trp* flies at intensities equivalent to ∼0.1 photons/microvillus/s (∼3000 photons/photoreceptor/s). The results also quantitatively confirm a similarly, and even slightly more severe phenotype, in *inaC* mutants lacking PKC. This indicates, as previously proposed (Gu et al., 2005), that Ca^2+^ influx, acting through Ca^2+^ (and DAG)-dependent PKC prevents PtdIns(4,5)*P*_2_ depletion by inhibition of PLC. An alternative, or complementary, interpretation of these results might be that Ca^2+^ (and PKC) facilitate PtdIns(4,5)*P*_2_ resynthesis; however, over a wide range of intensities, we could find no obvious effects of light adaptation (with associated Ca^2+^ influx and PKC activation) on the rate of resynthesis of PtdIns(4,5)*P*_2_ (Fig. S3). Nevertheless, it remains unclear how PKC inhibits PLC, given that PLC is not believed to be a direct substrate for PKC in the photoreceptors (Huber et al., 1996). One possibility is that it does so through the INAD scaffolding molecule, which is a PKC target (Voolstra et al., 2015), although an effect at the level of the G-protein cannot be excluded.

Ca^2+^-dependent inhibition of PLC was independently supported by the sensitive, and rapid, PtdIns(4,5)*P*_2_ depletion seen in the absence of any Ca^2+^ influx in *trpl*;*trp* double mutants. In fact, PtdIns(4,5)*P*_2_ was ∼1000-fold more sensitive to depletion in *trpl*;*trp* than in wild type, and ∼10-fold more sensitive than in *inaC* (or *trp*). This indicates an additional, eye-PKC-independent inhibition of PLC by Ca^2+^ that can also be mediated by the residual Ca^2+^ influx through TRPL channels (Fig. 4C). The mechanism for this is unknown: we cannot exclude a contribution of alternative PKC isoforms, but other possibilities might include charge interactions between the negatively charged PtdIns(4,5)*P*_2_ headgroup and Ca^2+^ (Wang et al., 2014), or the Ca^2+^-dependent facilitation of arrestin binding to active metarhodopsin (Liu et al., 2008).

The profound slowing of PtdIns(4,5)*P*_2_ and PtdIns4*P* resynthesis in *rgdA* and *rdgB* mutants confirm the essential involvement of DAG kinase and PITP proteins in PtdIns(4,5)*P*_2_ resynthesis. Significantly reduced *F*_max_ and *F*_max_*/F*_min_ ratios also suggest that the absolute level of PtdIns(4,5)*P*_2_ and PtdIns4*P* in the rhabdomeres of both these mutants was substantially reduced (∼2–3-fold). Despite the profoundly slowed recovery in these mutants, eventually some PtdIns(4,5)*P*_2_ was replenished in both *rdgA^1^* (a severe hypomorph) and *rdgB^2^* (reportedly a protein null), raising the possibility of alternative pathways for resynthesis. In this respect, it is perhaps important that a minor slow component of PtdIns(4,5)*P*_2_ recovery with a similar timecourse was seen in the wild-type background (Fig. 4C).

### PtdIns(4,5)*P*_2_ levels and sensitivity to light

Simultaneous measurement of Tb^R332H^ fluorescence and ERG in *trp* mutants showed remarkable quantitative equivalence with respect to both intensity dependence and recovery after decay (Figs 6 and 7) suggesting that the electrical light response in *trp*, which is mediated exclusively by TRPL channels, is essentially proportional to the level of PtdIns(4,5)*P*_2_. By contrast, the wild-type light response, which is dominated by TRP channels (Reuss et al., 1997), was relatively resistant to PtdIns(4,5)*P*_2_ depletion. On the one hand, this might imply differential sensitivity of TRP and TRPL channels to the (still unresolved) active products of PLC hydrolysis (putatively e.g. protons, mechanical forces, DAG or polyunsaturated fatty acids). On the other hand, an alternative (not mutually exclusive) explanation is that TRP (but not TRPL) channels are subject to a powerful non-linear Ca^2+^-dependent positive feedback (Reuss et al., 1997), which could be expected to result in full size ‘all-or-none’ single-photon responses even under conditions of reduced PtdIns(4,5)*P*_2_.

### Rate-limiting step

An unexpected finding was that the recovery of PtdIns4*P* was so fast (*t*½ ∼12 s in wild-type and 25 s in the *trp* mutant background), whereas PtdIns(4,5)*P*_2_ recovered with a halftime of only ∼45 s. This suggests that the rate-limiting step in the PtdIns(4,5)*P*_2_ cycle in the photoreceptors is the final step, that is, conversion of PtdIns4*P* into PtdIns(4,5)*P*_2_ by PtdIns4*P* 5-kinase (Chakrabarti et al., 2015). This is contrary to limited available data in mammalian cultured cells (Falkenburger et al., 2010; Willars et al., 1998), but was supported by measurements using Ci-VSP, which showed that recovery of the light response following PtdIns(4,5)*P*_2_ depletion (to PtdIns4*P*) by Ci-VSP followed a similar timecourse to the recovery from light-induced PtdIns(4,5)*P*_2_ depletion (Fig. 8). The functional significance of this slow final step is unclear, but it can be speculated that it might protect the photoreceptors from total depletion of all the phosphatidylinositol and PtdIns4*P* reserve in the rhabdomere under conditions of bright illumination.

In conclusion, after testing a range of fluorescently tagged lipid probes, we have identified Tb^R332H^ and P4M as suitable for real-time monitoring of PtdIns(4,5)*P*_2_ and PtdIns4*P* in *Drosophila* photoreceptors. Using appropriate paradigms, these can be used to track and quantify phosphoinositide breakdown and resynthesis in the rhabdomeres of completely intact living flies, with a precision we believe to be unsurpassed in other systems. This approach is straightforward to implement in the fly eye, and although Tb^R332H^ and P4M should prove valuable for further physiological and molecular dissection of this important signalling pathway, this methodology can be readily extended to other genetically encoded fluorescent probes.

## MATERIALS AND METHODS

### Flies

*Drosophila melanogaster* were reared in the dark at 25°C on standard (cornmeal, agar, yeast and glucose) diet. Although probes (see below) were expressed in a white-eyed (*w^1118^*) background, the mini-white gene used as a marker in the expression vector confers eye colour, which varies from pale orange to red between independent inserts of even the same transgene. Eye colour had little, if any, effect on normalised data; however, deeper eye colours have lower absolute fluorescence, and greater *F*_max_/*F*_min_ ratios due to enhanced contrast of rhabdomere fluorescence (Fig. S2). In principle, *F*_max_/*F*_min_ provides information on the extent of phosphoinositide depletion or its initial level, but such comparisons were only made between flies with the same transgene insert and eye colour. Some lines were also crossed into a *cn*, *bw* background, resulting in white eyes despite the mini-white transgene. Transgenes were crossed into the following mutant backgrounds: *norpA^P24^*, a null mutant for PLC (Bloomquist et al., 1988); *trp^343^*, a null mutant of the TRP channel (Scott et al., 1997); *trpl^302^*;*trp^343^* a null double mutant for both light-sensitive channels; *inaC^P209^*, a null mutant for protein kinase C (Smith et al., 1991); *rdgA^1^*, a severe hypomorph of DAG kinase (Masai et al., 1993); *rdgB^9^* (also known as *rdgB^KS222^*), a severe hypomorph; and *rdgB^2^*, a protein null allele of a phosphatidylinositol transfer protein (Vihtelic et al., 1993).

### Transgenic flies

cDNA for the PH domain of PLCδ1 (Stauffer et al., 1998) was subcloned into a plasmid containing the *trp* promoter. cDNAs for Tb^R332H^ (Quinn et al., 2008), OSH1, OSH2, P4M and Ci-VSP were obtained in mammalian vectors and subcloned into the pCaSper4 vector containing the *ninaE* promoter. Constructs were sequenced and confirmed at the sequencing facility of Department of Biochemistry, University of Cambridge, UK. Concentrated plasmid DNA preps (∼800–1000 ng/μl) were made using QIAfilter Plasmid Midi Kit and sent to the Department of Genetics, University of Cambridge, UK, or BestGene, Chino Hills, CA for generation of transgenic flies by p-element transformation. Both homozygotes (two copies of transgene) and balanced lines (one copy) were used, with at least two independent transgene inserts tested for each construct, with no obvious difference in results, apart from the brighter fluorescence and deeper eye colour when using two copies of the transgenes (Fig. S2).

### Imaging of dissociated ommatidia

Fluorescence from isolated ommatidia was viewed with a 40× NA 1.30 oil immersion objective on a Nikon Eclipse TE300 inverted microscope, using freshly dissociated ommatidia prepared as previously described for electrophysiological patch-clamp experiments (Hardie, 1991; Hardie et al., 2002). Images were captured, typically at 1–5 frames s^−1^, using an Orca 4.0 Flash camera (Hamamatsu). The average intensity was measured in ROIs in the rhabdomere and cytosol using Hamamatsu HC image software (e.g. Fig. 1). The bath contained (in mM): 120 NaCl, 5 KCl, 10 *N*-Tris-(hydroxymethyl)-methyl-2-amino-ethanesulphonic acid (TES), 4 MgCl_2_, 1.5 CaCl_2_, 25 proline and 5 alanine, pH 7.15. Chemicals were ordered from Sigma-Aldrich.

### Live imaging of the deep pseudopupil and calibration

Fluorescence from the DPP of intact flies was measured as previously described (Satoh et al., 2010). Briefly, flies were fixed by the head and proboscis with low-melting point wax in truncated pipette tips, mounted on a micromanipulator and observed with a 20×0.35 NA Fluor objective on the Nikon inverted microscope. The DPP from a frontal-dorsal eye region was cropped with a rectangular diaphragm (similar in size to the image frames in Fig. 2A) and sampled at up to 500 Hz using a photomultiplier tube (Cairn Research Ltd) collecting fluorescence excited by a blue (470 nm peak) ultrabright LED (Cairn Research Ltd) and imaged or measured through a 515 nm dichroic and OG515 long pass filters. Fluorescence signals were sampled and analysed using pClamp10 software (Molecular Devices) and, unless otherwise stated, normalised between *F*_max_ (1.0) and *F*_min_ (0.0). Photo-reisomerisation of M into R was achieved by long-wavelength light delivered by an ultrabright orange–red LED (640 nm, Thorlabs) via the microscope eyepiece. Calibrated green illumination (∼545 nm±50 nm), also delivered via an eyepiece adapter, and came from a white ultrabright LED filtered by a GG495 nm longpass filter (Fig. S4).

Fly rhodopsin (R) absorbs maximally at 480 nm, and the metarhodopsin state (M) at ∼570 nm. The two states are thermostable, photo-interconvertible and exist in a photoequilibrium determined by their photosensitivity spectra and the spectral content of illumination (Fig. S4; and see Minke and Kirschfeld, 1979). Photoequilibration was achieved within <100 ms for the blue excitation LED (generating ∼70% M, 30% R) and 1–2 s for the orange–red LED (generating ∼2% M, 98% R). The intensity of the green light used for determining the intensity dependence of phosphoinositide depletion was calibrated by measuring the rate at which it photoisomerised M into R. Long-wavelength light reflected and scattered back out of the eye is more effectively absorbed by M than it is by R. Consequently, when delivered after photoequilibration with blue light (generating ∼70% M), the intensity of back-scattered long-wavelength light increases as M is photoreisomerised into R with an exponential timecourse that provides a direct measure of the rate of photoisomerisation of M into R (Fig. S4). The number of M absorptions per second per microvillus was calculated from this, assuming there are 1000 rhodopsin molecules per microvillus (Harris et al., 1977). The relative absorption of R by the same light (∼6 times less) was calculated by convolving the spectral distribution of the light (measured with an Ocean Optics spectrometer) with photosensitivity spectra of R and M states of the pigment, and correcting for the difference (Fig. S4).

### Electrophysiology

Electroretinograms (ERGs) were recorded as described previously (e.g. Satoh et al., 2010) from flies immobilised with low-melting point wax in truncated pipette tips, as for optical recordings, using low-resistance (∼10 MΩ) glass microelectrodes filled with fly Ringer (140 mM NaCl, 5 mM KCl, 1.5 mM CaCl_2_, 4 mM MgCl_2_) inserted into the eye. When recording ERGs alone, the indifferent electrode was a similar electrode inserted into the head capsule near the ocelli. For simultaneous fluorescence and ERG recordings, the indifferent electrode was a 50 µm chloridised silver wire contacting the head through a drop of ECG-conducting gel. Signals were amplified by a Neurolog NL102 DC (Digitimer) or a DAM60 preamplifier (World Precision Instruments) and sampled and analysed using pClamp software (Molecular Devices CA). Whole-cell patch clamp recordings of dissociated ommatidia were made as previously described (Hardie et al., 2001) using 10–15 MΩ patch pipettes containing (in mM) 140 K gluconate, 4 MgATP, 1 NAD, 0.4 NaGTP and 10TES with the bath solution described above.
